# Mental practice modulates functional connectivity between the cerebellum and the primary motor cortex

**DOI:** 10.1016/j.isci.2022.104397

**Published:** 2022-05-13

**Authors:** Dylan Rannaud Monany, Florent Lebon, William Dupont, Charalambos Papaxanthis

**Affiliations:** 1 INSERM UMR1093-CAPS, Université Bourgogne Franche-Comté, UFR des Sciences du Sport, F-21000 Dijon, France

**Keywords:** Biological sciences, Neuroscience, Sensory neuroscience

## Abstract

Our brain has the extraordinary capacity to improve motor skills through mental practice. Conceptually, this ability is attributed to internal forward models, which are cerebellar neural networks that can predict the sensory consequences of motor commands. In our study, we employed single and dual-coil transcranial magnetic stimulations to probe the level of corticospinal excitability and cerebellar-brain inhibition, respectively, before and after a mental practice session or a control session. Motor skill (i.e., accuracy and speed) was measured using a sequential finger tapping-task. We found that mental practice enhanced both speed and accuracy. In parallel, the functional connectivity between the cerebellum and the primary motor cortex changed, with less inhibition from the first to the second. These findings reveal the existence of neuroplastic changes within the cerebellum, supporting the involvement of internal models after mental practice.

## Highlights

• The update of internal forward models involves cerebellar neural adaptations
• Mental practice is assumed to engage internal forward models
• Cerebellar-brain Inhibition was probed by TMS before and after mental practice
• Mental practice reduces Cerebellar-brain Inhibition and may update internal models

## Graphical abstract

**Figure:**
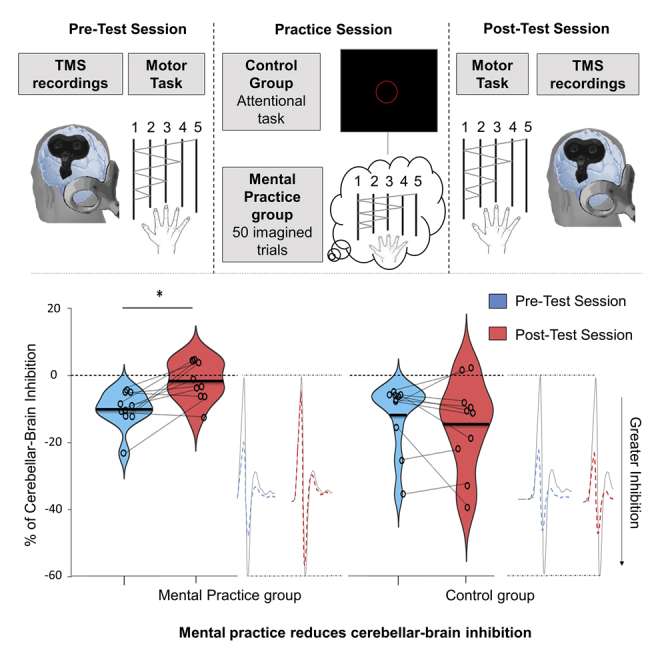

## Teaser

Biological sciences; Neuroscience; Motor neuroscience

## Introduction

A remarkable feature of our brain is its ability to create mental images of past and future events. Part of this mental process is motor imagery, i.e., the internal simulation of body movements without execution (Ruffino et al., 2021). Professional athletes, dancers, and musicians, as well as patients with sensorimotor deficits use mental practice to improve their motor performance (Schuster et al., 2011, for review). The concept of internal forward models offers the theoretical basis to understand the mental practice process and the associated changes in motor behavior (Kilteni et al., 2018). An internal forward model is a neural network that mimics the causal flow of the physical process by predicting the future sensorimotor state (e.g., position and velocity) given the goal of the movement, the efferent copy of the motor command, and the current state (Wolpert and Flanagan, 2001). Both executed and mental movements seem to share this process. During movement execution, predictions are compared with sensory feedback. Any discrepancy constitutes an error signal that can update the internal forward model (i.e., better predictions) and the controller (i.e., better motor commands). During motor imagery, such comparison is not possible as no movement is produced. However, the goal of the action (e.g., a specific dancing figure) is compared with the prediction from the forward model (i.e., how the dancing figure would be if executed). Any difference between the prediction and the goal acts as an internal error signal that can update and improve the controller and the forward models via a “self-supervised process” (Gentili et al., 2010).

Neurophysiological and clinical studies consider the cerebellum as a potential locus of internal forward models (Honda et al., 2018; Izawa et al., 2012). Update of motor predictions and motor commands would imply — among others — neuroplastic changes of the neural pathways between the cerebellum and cortical motor areas (Celnik, 2015). Dual coil transcranial magnetic stimulation (TMS) is a particularly appropriate method to indirectly probe cerebellar adaptations by measuring the influence exerted by the cerebellum onto the primary motor cortex (M1). Specifically, the indirect inhibitory influence of Purkinje cells onto M1 (called cerebellar-brain inhibition or CBI) decreases after motor practice (Baarbé et al., 2014). Intriguingly, although behavioral studies confirm the positive effects of mental training in motor learning through forward model predictions, neural evidence supporting them is missing.

## Results

**Figure 1:**
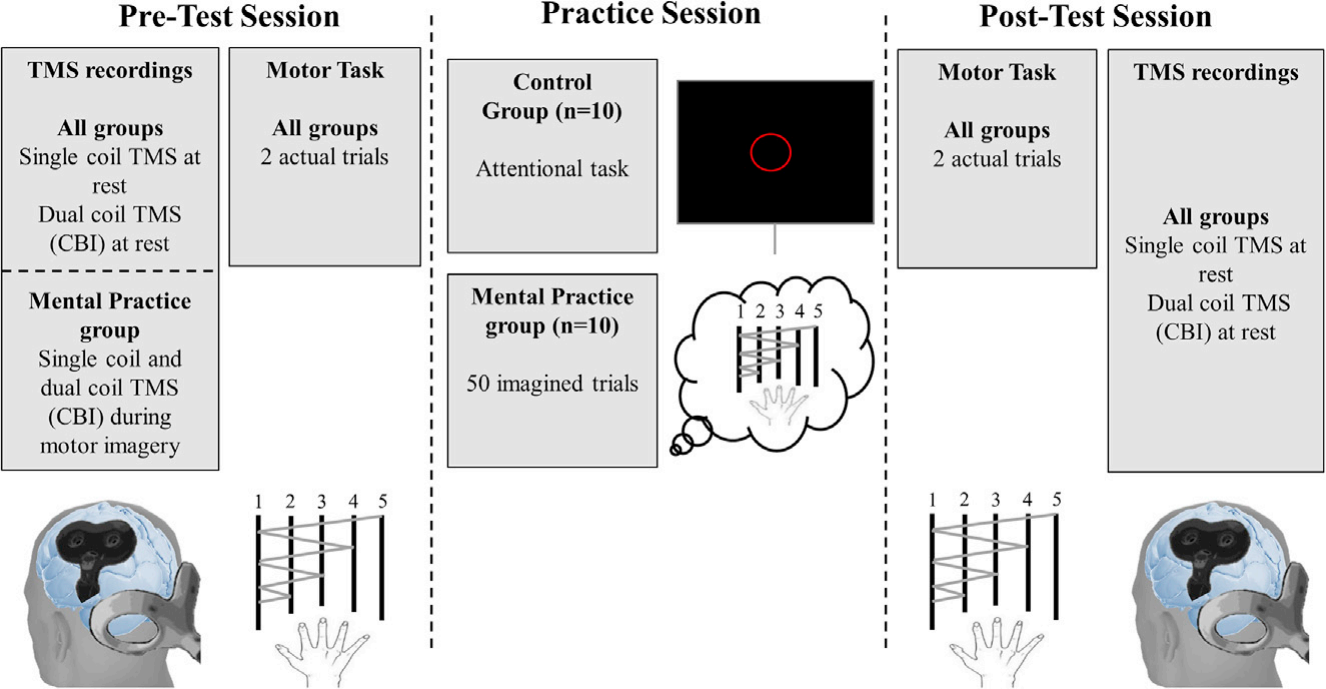
Schematic representation of the experimental procedure At pretest and postttest sessions, both groups executed two trials of the sequential finger-tapping task (order: 1 – 2 – 1 – 3 – 1 – 4 – 1 – 5) with their right hand, as fast and accurately as possible. Each number corresponds to a digit (1 = thumb; 5 = pinkie). Each trial lasted 10 s. Movement speed was defined as the total number of executed sequences per trial, independent of their accuracy. Accuracy was defined as the total number of correct sequences achieved per trial. Corticospinal excitability and cerebellar-brain inhibition (CBI) were also assessed in both groups before (Pre-Test) and after (Post-Test) actual trials. Neurophysiological measurements were made at rest, i.e., the participants remained quiet without performing any task. In addition, CBI was probed during imagined movements for the mental practice group at Pre-Test. During the training session, the control group performed an attentional task, consisting of counting and memorizing a given number of red circles interspersed within white circles. The mental practice group performed 5 blocks of 10 imagined trials. The duration of both tasks was equivalent.

Here, we investigated whether improvement in motor performance after mental practice involves neural changes within the cerebellum. We designed an experiment (Figure 1) in which motor skill in a sequential finger-tapping task and CBI were tested before and after an acute session of mental practice (MP group) or of an attentional task (Control group). Movement speed (total number of executed sequences) and accuracy (number of correct sequences) were the markers of motor performance (Walker et al., 2003).

### Movement speed and accuracy

**Figure 2:**
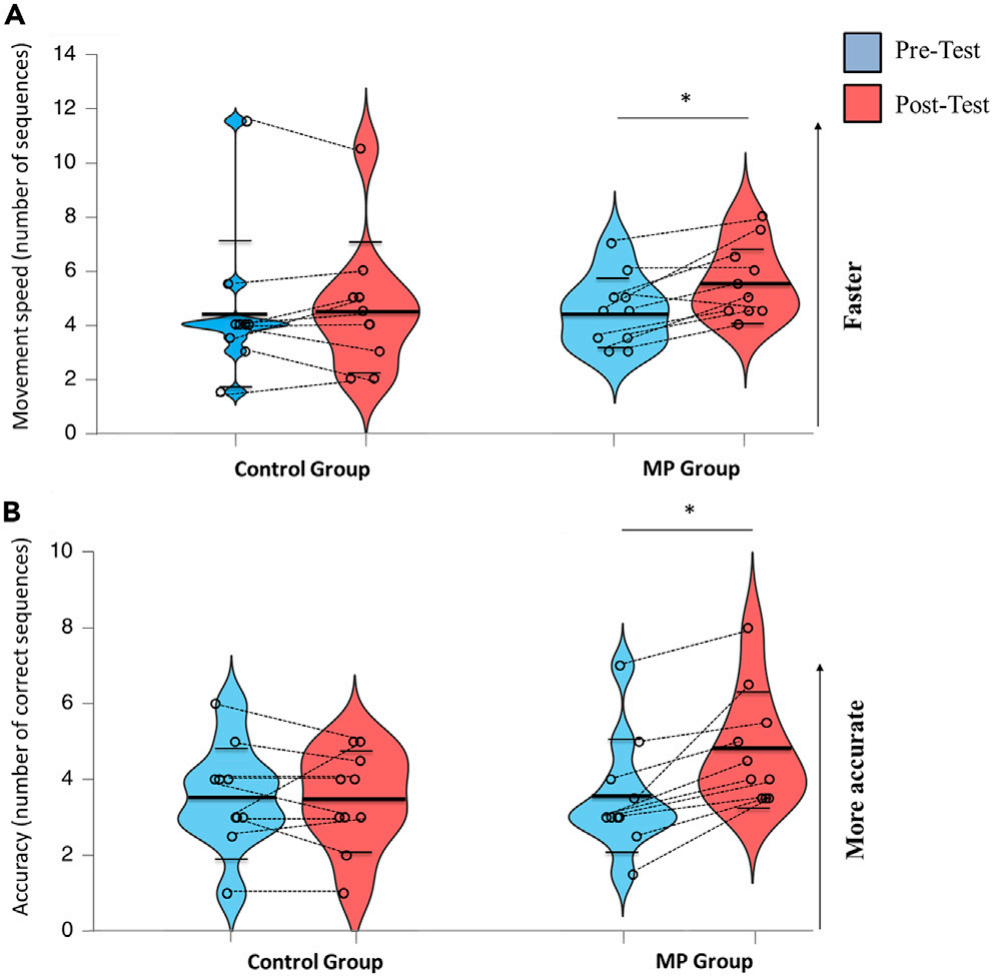
Movement speed and accuracy (A and B) Violin plots for movement speed (i.e., the total number of executed sequences, [A]) and accuracy (i.e., the number of correct sequences, [B]) for both groups at Pre-Test and Post-Test. Thick and thin horizontal lines mark mean and SD, respectively. Dots represent individual data per condition. Both parameters significantly increased following mental practice (MP) but not after the attentional task (control group). ∗: p < 0.05 (pairwise comparisons).

We found a Group∗Time interaction for both movement speed (*F*1,18 = 5.88, p = 0.026, *ηp2* = 0.25) and accuracy (*F*1,18 = 13.09, p < 0.01, *ηp2* = 0.42). Post hoc pairwise comparisons with Bonferroni corrections confirmed that the MP group was faster (Figure 2A) and more accurate (Figure 2B) at the sequential finger-tapping task after training (Pre-Test versus Post-Test; all *p’s* < 0.01). The same comparisons were not significant for the Control group (all *p’s* > 0.5). Note that we control for potential between groups’ differences at Post-Test while bringing the Pre-Test performances as a covariate (see Data S1, section ”Ancova for movement speed, accuracy, and DualRest”). It is worth noting that fingers’ muscles remained quiescent during mental practice (see Data S1, section “EMG during mental practice”), excluding any potential influence of muscle activation in skill improvement. However, both groups showed a comparable increase in mental fatigue after training (see Data S1, section ”Mental fatigue”).

### Cerebellar-brain inhibition

**Figure 3:**
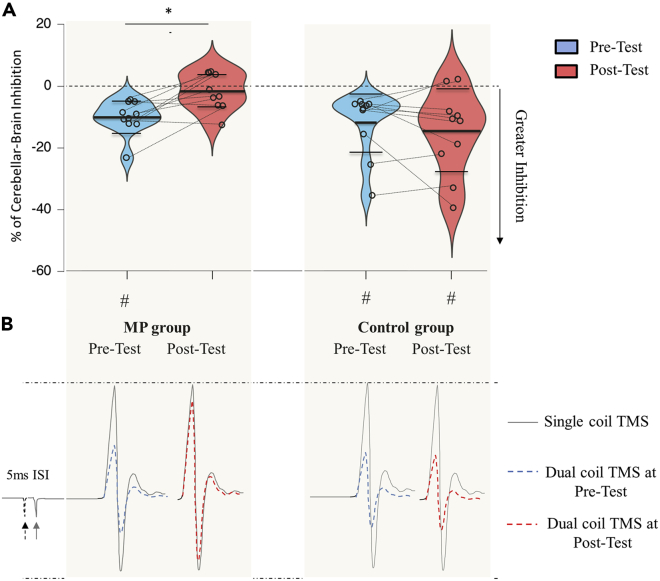
Cerebellar-brain inhibition (A) Violin plots for the percentage of CBI at pretest and posttest sessions for mental practice (MP) and control groups. Thick and thin horizontal lines mark mean and SD, respectively. Dots represent individual data per condition. The main finding is a disinhibition of CBI following MP. We used one-sample *t*-test against 0 to ensure the presence of CBI for the Control group at Pre-Test: (Mean ± SD: −11.84 ± 10.41%, *t*(9) = -3.59, p < 0.01, *Hedges’s g* = −1.02) and Post-Test (Mean ± SD: −14.71 ± 13.59, *t*(9) = -3.42, p < 0.01, *Hedges’s g* = −0.97). For the MP group, CBI values were different from 0 at Pre-Test (Mean ± SD: −9.92 ± 4.48%, *t*(9) = -5.728, p < 0.01, *Hedges’s g* = −1.63) but not at Post-Test, indicating no inhibition (Mean ± SD: −1.45 ± 5.87%, p = 0.45). ∗: p < 0.05 (pairwise comparisons); #: p < 0.05 (comparison to 0). (B) Illustration of motor-evoked potential (MEP) modulation. The interstimulus interval (ISI) between the conditioning pulse (black dotted arrow) and the test pulse (gray arrow) was 5 ms. Single-coil TMS over M1 (test pulse only) elicited an MEP in the target muscle (gray lines). When conditioning M1 with cerebellar stimulation, the MEP amplitude reduced, reflecting CBI (Pre-Test, blue dotted lines). Although the conditioned MEP remained reduced for the control group, it increased following MP showing a disinhibition mechanism (Post-Test, red dotted lines).

To probe neural changes within the cerebellum, we measured CBI between the right cerebellum and left M1 using dual-coil TMS. Note that before testing CBI, we first verified that the sole stimulation at the cervicomedullary junction did not elicit descending volleys into the spinal cord (see Data S1, section “Cervicomedullar output”) and that the TMS parameters were similar between the two groups (see Data S1, section “Rest motor threshold, MEPtarget, and CS intensity”). Thereafter, we found a modulation of CBI following mental practice, attested by the significant Group∗Time interaction (*F*1,18 = 8.42, p < 0.01, *ηp2* = 0.32, Figure 3). Interestingly, pairwise comparisons with Bonferroni corrections revealed that CBI was no longer present after practice for the MP group (Mean ± SD: −9.92 ± 5.48% at Pre-Test versus −1.45 ± 5.87% at Post-Test, p = 0.039), whereas it was still observed after the attentional task for the control group (Mean ± SD: −11.84 ± 10.41% at Pre-Test versus −14.71 ± 13.58% at Post-Test, p = 1).

It is worth mentioning that the disinhibition was because of plastic changes that occurred within the cerebellum. Indeed, corticospinal excitability at rest (single-coil TMS condition) was not modulated (see Data S1, section “Corticospinal excitability [Single-coil TMS]”), excluding neural adaptations between M1 and the target muscle after an acute session of mental practice. In addition, the disinhibition observed at rest after practice can be directly attributed to CBI modulation occurring during imagined movements (see Figure S1). Indeed, we found a significant reduction of CBI while imagining compared to rest at Pre-Test (Mean ± SD: −3.57 ± 7.07% and −9.92 ± 5.48%; *t*(9) = 3.06, respectively, p = 0.01, *Hedges’s g* = 0.96).

## Discussion

We provided evidence that an acute session of mental practice improves motor performance and induces neural adaptations within the cerebellum (i.e., reduction of CBI). These results reinforce the premise regarding the positive contribution of internal movement prediction during consecutive imagined actions. The state predictions (e.g., position and speed) generated by the forward model during mental practice may be appropriate to elicit functional adaptations within the corticocerebellar loop and thus improve motor skills (i.e., faster and more accurate movements). Clinical studies support this statement, showing that cerebellar lesions affect the motor imagery process of complex motor sequences and alter the update of internal movement predictions (Battaglia et al., 2006; Synofzik et al., 2008; Saunier et al., 2021). We would explain the reduction of CBI during mental practice by the long-term depression-like plasticity phenomenon within the cerebellum, as suggested for actual practice (Galea et al., 2011; Ishikawa et al., 2016; Tanaka et al., 2020). During actual movements, the inferior oliva compares inhibitory signals from deep cerebellar nuclei (sensory prediction) and excitatory signals from M1 (desired movement) and sensory feedbacks. The imbalance between these signals constitutes an error signal that induces long-term depression-like plasticity at the level of purkinje cell fibers. The reduction of the inhibitory output from the GABAergic purkinje cells onto deep cerebellar nuclei would indirectly increase M1 excitability. Notably, another comparison occurs between desired and predicted states at the level of deep cerebellar nuclei, constituting an internal feedback loop that adjusts motor commands before movement onset. During mental practice, we speculate that the inferior oliva only compares sensory prediction and desired movement, represented by inhibitory cerebellar signals and excitatory M1 signals, respectively. The excitatory inputs from sensory feedbacks are absent as no movement occurs during motor imagery. The internal feedback loop to adjust motor commands before onset of imagined movements might also be at play during mental practice. Further investigations are necessary to determine which neuroplastic mechanisms reduce CBI after mental practice.

### Limitations and conclusion

The current work contains some limitations. First, it should be stated that the current results must be considered with some caution. Indeed, the small sample sizes used in this study (10 participants per group) are likely to induce effects’ sizes overestimation bias (Kühberger et al., 2014). This can be legitimately assumed when considering the magnitude of the reported effects’ sizes. We suggest that further studies may minimize both overestimation bias and recruitment difficulties (owing to the high discomfort generated by cerebellar stimulations) by testing the effects of mental practice on cerebello-cortical neural adaptations with a within-subject design. Although dual-coil TMS is used to probe modulations of functional connectivity between the cerebellum and M1 (Spampinato et al., 2020), it is worth noting that the cerebellar stimulation may also reach nonmotor areas within the cerebellum, which could affect prefrontal-projecting cerebellar networks (Hardwick et al., 2021). Implementation of protocols aiming to disentangle these processes by using triple-coil TMS (i.e., cerebellum, prefrontal cortex, and M1, Pauly et al., 2021) or by modulating the current flow (e.g., anteroposterior) and the ISI could be beneficial on that point. In addition, we acknowledge that more recent and adapted methods, such as threshold-hunting techniques, could have been used to determine the rest motor threshold (Kallioniemi et al., 2021). Finally, it is worth noting that a supplementary control task, involving motor imagery of movements that are unrelated to the practiced motor sequence, could contribute to control for task-specificity effects.

In conclusion, the current findings suggest the importance of the corticocerebellar loop in motor learning through mental practice, which can be used in isolation or in addition to actual practice to improve motor performance in healthy individuals or patients with motor deficits. Our results corroborate and extend those of previous studies in skill learning (Spampinato and Celnik, 2017), which showed a significant reduction of CBI immediately after an acute session of physical training. Despite the absence of sensorimotor feedback during mental practice, the cerebellum is at play as part of the internal forward model to predict the sensory consequences of the imagined action and to adapt motor commands via a putative self-supervised process.

## STAR★Methods

### Key resources table

**Table:** 

REAGENT or RESOURCE	SOURCE	IDENTIFIER
**Deposited data**		

Analyzed data	This paper	https://doi.org/10.17605/OSF.IO/7238X

### Resource availability

#### Lead contact

Further information and requests for resources should be directed to and will be fulfilled by the lead contact, Florent Lebon (Florent.Lebon@u-bourgogne.fr).

#### Material availability

This study did not generate new unique reagents.

### Experimental model and subject details

#### Participants

Twenty healthy right-handed volunteers participated in the study (4 women, mean age: 22.75 years old, range: 20–26). All were screened for contraindications to TMS by a medical doctor and had a normal or corrected vision. The participants were randomly assigned to the mental practice group (MP group, n = 10, mean age: 22,7, range 20–26) and the Control group (n = 10, mean age: 22.8, range 20–25). The study was approved by the CPP SOOM III ethics committee (ClinicalTrials.gov Identifier: NCT03334526) and complied with the standards set by the Declaration of Helsinki (Version, 2013; excluding pre-registration). Informed consent was obtained from all participants.

### Method details

#### Behavioral task

The motor task was a computerized version of the sequential finger-tapping task (Karni et al., 1998; Debarnot et al., 2009). The participants were comfortably seated in front of a customized keyboard and performed a sequence of eight movements using their right fingers in the following order: 1-2-1-3-1-4-1-5 (1: thumb, 2: index finger, 3: middle finger, 4: ring finger, 5: pinky), as fast and accurately as possible (Figure 1). At Pre-Test and Post-Test sessions, the participants performed two trials of 10s each. Between the test sessions, the MP group imagined the same sequence during five blocks of ten trials (total number of trials = 50). Each trial lasted 10 s with 10-s rest. The participants of the MP group placed their right hand on the keyboard, and imagined the motor sequence with the following instructions: “*try to imagine yourself performing the motor task as fast and accurately as possible, by feeling your fingers moving as if you were moving it*”. Electromyographic (EMG) activity was recorded during mental practice to ensure the absence of muscular activity. The Control group performed a visual recognition task, during which the participants counted and memorized the number of red circles interspersed within white circles. The number of red circles varied between blocks to avoid habituation effects. The participants reported the number of red circles they memorized after each block to confirm that they were focused on the task. The duration of this task matched the duration of mental practice. For both groups, we assessed the mental fatigue before and after the tasks, using a 10-cm visual analog scale (0 cm: ‘no fatigue’, 10 cm ‘maximal fatigue’).

#### Transcranial magnetic stimulation

We assessed the level of corticospinal excitability with single-coil transcranial magnetic stimulation (TMS) and the amount of cerebellar-brain inhibition (CBI) with dual-coil TMS. Single-coil and dual-coil TMS were applied using monophasic Bi-Stim^2^ stimulators (*The Magstim Co., Whitland, UK*). Motor-evoked potentials (MEPs) were recorded in the right Abductor Pollicis Brevis (APB) muscle.

##### TMS over M1 (test stimulus, TS)

Single-coil stimulations were applied with a 70-mm figure-of-eight coil placed over the left M1 in a posterior position at 45° from the sagittal plane to induce a postero-anterior current flow. We delivered stimulations at the level of the primary motor cortex to visually identify the site that elicits the highest and most consistent MEPs amplitude in APB. The presumed hotspot was then marked with a pen on the scalp. A series of stimulation was then elicited around the hotspot in the form of a grid (about 0.5 cm distance between each point) to ensure that our location was as adequate as possible. If it was not the case, the process was repeated on another spot. We determined the rest motor threshold (rMT) as the lowest stimulator output that elicited four of eight MEPs with peak-to-peak amplitude equal to or greater than 0.05 mV (Stinear and Byblow, 2004; Schecklmann et al., 2020). Then, we determined the intensity to evoke MEP_max_ at rest, i.e., the individual highest peak-to-peak MEP amplitude. To that purpose, 8 stimulations were administered at 140 and 150% of the rMT. We chose to begin at such values in order to reduce both the duration of the session and the number of stimulations, and because previous literature showed that such stimulation intensities are likely to induce MEPs that approach or correspond to the TMS recruitment curve’s plateau (Pellegrini et al., 2018). The obtained MEP values were then averaged and compared to check if the plateau was reached. Specifically, we sought to obtain less than 10% of amplitude variation between stimulation conditions. If so, the highest averaged value between both was considered as MEP_max_. If not, the operation was reiterated after eight stimulations at 160% of the rMT. For the rest of the experiment, we considered MEP_target_ as half of MEP_max_ amplitude (Pitcher et al., 2015; Neige et al., 2020). MEP_Target_ was set at 124.8 ± 11.52% and 123.39 ± 12.61% (mean ± SD) of rMT for Control and MP groups, respectively. All single-coil stimulations were manually administered with 5s-to-7s intervals.

##### TMS over the cerebellum (conditioning stimulus, CS)

We used a double-cone coil (wing diameter: 110 mm) to stimulate deep cerebellar structures. The double-cone coil was positioned over the cerebellum on the horizontal line between the inion and the external auditory meatus at 2 cm right to the inion. The stimulation intensity was fixed at 150% of individual M1 rMT. Although the cerebellar stimulation intensity in the current experiment was lower than that used in previous studies (Bunday et al., 2014), we delivered five cerebellar stimulations alone at rest at Pre-Test to verify the absence of descending volleys in EMG traces.

##### Dual-coil stimulations (CS and TS)

Dual-coil stimulations were applied through the combination of the double-cone coil TMS (CS) over the right cerebellum and a 70-mm figure-of-eight coil over left M1 (TS, see above). Dual-coil stimulations were triggered by software developed in our laboratory. The inter-stimulation interval between CS and TS was set to 5 ms to ensure the activation of cerebellar-M1 inhibitory pathways (Fisher et al., 2009) and the interval between paired stimulations was set to 10s.

#### Adaptive threshold-hunting technique

This method has been recently developed to overcome the potential limitations of conventional paired-pulse TMS protocols, such as large variability in MEP amplitude and a ‘‘floor/ceiling effect” when the observed inhibition leads to complete MEP suppression (Cirillo and Byblow, 2016; Van der Bos et al., 2018). The hunting-threshold was defined as the TS intensity (expressed in percentage of the maximal stimulator output - %MSO) required to elicit the MEP_target_ in the relaxed APB. To do so, we used an online freeware (TMS Motor Threshold Assessment Tool, MTAT 2.0), based on a maximum-likelihood Parameter Estimation by Sequential Testing. Following previous studies, the software has been parameterized with assessment without *a priori* information (Cirillo et al., 2018; Neige et al., 2020). Once the amplitude of MEP_target_ and the intensity of CS (150% rMT) were set, we determined the TS intensity (%MSO) required to maintain the MEP_target_ amplitude across the experimental conditions. Therefore, the TS intensity was the dependent variable of the experiment.

Twenty stimulations were delivered for the single-coil TMS condition (SingleRest) and twenty stimulations for the dual-coil TMS condition (DualRest) at rest in the Pre-test and Post-Test sessions. In addition, the same number of stimulations was derived during single-coil TMS (SingleMI) and dual-coil TMS (DualMI) during motor imagery for the MP group only at Pre-Test. The order of the stimulation conditions was randomized across participants.

#### Experimental procedures

The experimental protocol included two test sessions (Pre-Test and Post-Test) and a training session. Pre-Test and Post-Test included both neurophysiological (single- and dual-coil TMS) and behavioral (sequential finger tapping task) recordings. The practice session consisted of mental practice or an attentional task. During Pre- and Post-Test, we determined the required intensity to obtain MEP_target_ by means of single- and dual-coil TMS at rest for both groups and during motor imagery of a maximal voluntary contraction of APB for MP group only. To assess motor skill, we measured the movement speed and accuracy of two actual trials of the finger sequence. It is worth noting that the two trials were performed after and before the TMS measurements at Pre-Test and Post-Test, respectively. During the training session, the participants were assigned, depending on their group, to a mental practice session (50 imagined trials) or an attentional task, consisting in counting and memorizing a given number of red circles interspersed within white circles.

#### Motor imagery questionnaire

Participants of the MP group were asked to complete the French version of the Motor Imagery Questionnaire to assess self-estimation of their motor imagery vividness (Loison et al., 2013). For this questionnaire, the minimum score is 8 (low imagery vividness) and the maximum one is 56 (high imagery vividness). In the current study, the average score of the MP group participants (Mean ± SD: 43.6 ± 5.04) suggests good motor imagery vividness.

### Quantification and statistical analysis

#### Sample size estimation

First, we estimated a large effect size (*ηp2* = 0.2, *Cohen’s f* = 0.4) from a previous similar study. Specifically, we used F (4.617) and df (_1,18_) from a 2 × 2 interaction in Baarbé et al. (2014) (Pre-Intervention vs Post-Intervention and Experimental group versus Control Group). With such an effect size and a power of 0.8, we estimated that 8 participants per group would be needed, using G∗ Power (*version 3.1.9.4.,* Faul et al., 2007). We finally chose to increase our sample sizes to 10 participants per group to be consistent with recent studies in the field (Spampinato and Celnik, 2017).

#### Behavioral parameters

Two motor parameters were assessed: movement speed and accuracy. These parameters were extracted for each trial and averaged for Pre-Test and Post-Test. Movement speed was defined as the total number of executed sequences per trial, independently of their accuracy. Accuracy was defined as the total number of correct sequences executed per trial.

#### Neurophysiological parameters

Values were quantified as %MSO for the four TMS recordings conditions (i.e., SingleRest, SingleMI, DualRest, and DualMI). To investigate variations of Cerebellar-Brain Inhibition at rest and during MI in dual-coil TMS conditions, we used the following formula:

$$\text{Dual\%}=\frac{\left(Single\;coil\;TMS\right)-\left(Dual\;coil\;TMS\right)}{\left(Single\;coil\;TMS\right)}\times 100$$

where positive values indicate facilitation and negative values indicate inhibition. DualRest and DualMI were normalized according to SingleRest and SingleMI, respectively.

#### Electromyographic recording and analysis

EMG recordings of the right APB muscle were made with surface Ag/AgCl electrodes in a belly-tendon montage. A ground electrode was placed on the styloid process of the ulna. The EMG signals were amplified and band-pass filtered (10–1000 Hz, Biopac Systems Inc.) and digitized at a sampling rate of 2000 Hz for offline analysis. Background EMG was monitored for the 100 ms preceding every TMS pulse to ensure a complete muscle relaxation (i.e., EMG below 0.02 mV, Mizuguchi et al., 2011) throughout the experiments, using the following formula:

$$RMS=\sqrt{\frac{1}{MD}\int_{0}^{MD}{\left(EMG\right)}^{2}dt}$$

#### Statistical analysis

Statistical analysis was performed using STATISTICA (*13.0 version; Stat-Soft, Tulsa, OK*). Normality was checked before inferential analysis using Shapiro Wilk tests. Hedges’s g (t-tests) and partial eta squared were reported to provide information on effect sizes for t-tests and mixed ANOVA, respectively. p-values were adjusted accordingly using the Bonferroni method when several tests were performed on the same variable. The threshold of statistical significance was set to α = 0.05.

First, movement speed and accuracy were analyzed using a mixed ANOVA analysis with Time (Pre-Test vs. Post-Test) as a within-subject factor and Group (Control vs. MP) as a between-subject factor. Post-hoc pairwise comparisons with Bonferroni corrections were performed in case of significant Group∗Time interaction. A Friedman’s ANOVA(normality was violated) was performed to compare the EMG activity of APB at each imagined block with recording at rest, to ensure that muscles remained silent during MP.

Then, DualRest was analyzed using a GLM analysis with Time (Pre-Test versus Post-Test) as a within-subject factor and Group (Control versus MP) as a between-subject factor. Pairwise comparisons with Bonferroni corrections were performed in case of significant Group∗Time interaction. One-sample t-tests versus 0 were used to test if dual-coil TMS induced a significant amount of CBI at Pre-Test and Post-Test for Control and MP groups. Also, to test whether the cerebellum is involved during motor imagery before practice (Tanaka et al., 2018), we performed a one-tailed paired-sample t-test opposing DualRest and DualMI for the MP group at Pre-Test.

All statistical details of experiments can be found in the Results section, in the Data S1, in figures and in figures legends. Results of one-sample t-tests and pairwise comparisons are reported in the main figures using the symbols “#” and “∗”, respectively. The symbol “§” is used in Figure S1 to report a paired t-test effect. Reported data are expressed as mean ± SD.

## Data Availability

• Anonymized data have been provided at Open Science Framework and are publicly available as of the date of publication. The DOI is listed in the Key resources table. • This paper does not report original code. • Any additional information required to reanalyze the data reported in this paper is available from the Lead contact upon request.

## Supporting citations

The following references appear in the Supplemental information: Rozand et al. (2016).
