# Supplementary material for: Mental practice modulates functional connectivity between the cerebellum and the primary motor cortex
Source: iScience. 2022 May 13;25(6):104397. doi: 10.1016/j.isci.2022.104397 (PMC9142644; doi:10.1016/j.isci.2022.104397)
Supplement: Document S1. Figures S1 [file mmc1.pdf]

iScience, Volume *25*

Supplemental information

Mental practice modulates functional connectivity between the cerebellum and the primary motor cortex

Dylan Rannaud Monany, Florent Lebon, William Dupont, and Charalambos Papaxanthis

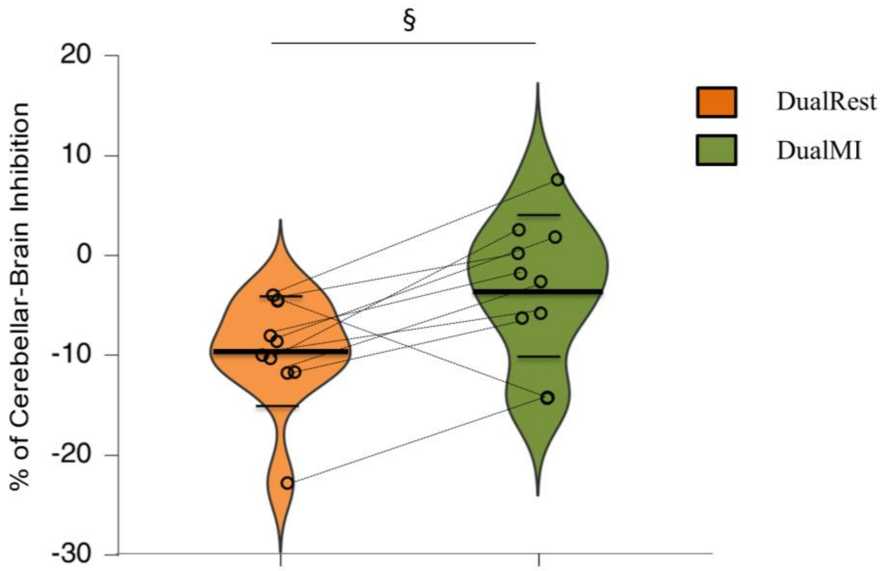

Supplemental Figure 1: Violin plots for the percentage of Cerebellar Brain inhibition at rest (DualRest, on the left) and during motor imagery (DualMI, on the right) of a maximal contraction of the APB muscle at Pre-Test, related to Figure 3. Thick and thin horizontal lines mark mean and SD, respectively. Dots represent individual data per conditions. §: $p<0.05$ (paired t-test).
