# Supplementary material for: Mental practice modulates functional connectivity between the cerebellum and the primary motor cortex
Source: iScience. 2022 May 13;25(6):104397. doi: 10.1016/j.isci.2022.104397 (PMC9142644; doi:10.1016/j.isci.2022.104397)
Supplement: Document S2. Data S1 [file mmc2.docx]

**COMPLEMENTARY RESULTS**

***Ancova for movement speed, accuracy, and DualRest***

To control for the “Time” factor, we performed an Ancova analysis, opposing the Post-Test of Control and MP groups with Pre-Test as a covariate, considering movement speed, accuracy and DualRest. Results of Ancova are coherent with those of the mixed-Anova, suggesting differences between Control and MP groups for movement speed (*F*_1, 17_=6.27, *p*=0.023, *η_p_^2^*=0.27), accuracy (*F*_1, 17_=14.07, *p*<0.01, *η_p_^2^*=0.45) and DualRest (*F*_1, 17_= 9.1, *p*<0.01, *η_p_^2^*=0.35).

***EMG during mental practice***

We used the Wilcoxon test (normality was violated) to verify if EMG activity increased during mental practice. Precisely, we compared the RMS of APB at each imagined block with the RMS recorded at rest. The grand average of EMG activity was 0.01 mV ±0.01 during mental practice and comparable to that recorded at rest (0.009 mV ±0.01). The statistical comparison revealed no significant difference for each block (for all *Z*< 1.27; *p*> 0.2), meaning that the MP group did not significantly activate muscles during mental practice.

***Mental fatigue***

We used a mixed ANOVA analysis with Time (Pre-Test vs. Post-Test) as a within-subject factor and Group (Control vs. MP) as a between-subject factor to test if mental fatigue increased after mental practice or attentional task (Rozand et al., 2016). There was a main effect of Time (*F*_1,18_=13.77, *p*<0.01, *η_p_^2^*=0.41), without main effect of Group (*F*_1,18_=1.74, *p*=0.2) nor Group*Time interaction (*F*_1,18_=0.002, *p*=0.96). The main effect of Time is explained by a global increase of mental fatigue between Pre-Test and Post-Test, independently of the task (MP group; Pre-Test: 3.25 ±1.37 cm, Post-Test: 3.63 ±1.45 cm, difference: +0.38 ±0.49 cm, Control group; Pre-Test: 2.34 ±1.65 cm, Post-Test: 2.71 ±1.76 cm, difference: +0.37 ±0.41 cm). This suggests that mental fatigue increased after both mental practice and attentional tasks.

***Cervicomedullar output***

We used one-tailed one-sample t-tests against the reference value 0.05 to ensure that the amplitude of raw EMG following cerebellar stimulations alone remained significantly lower than the rest motor threshold (0.05 mV). We found that EMG traces remained below the rest motor threshold for the Control group (0.02 ±0.02 mV; *t*(9)=-4.61, *p*<0.01, *Hedges’s g*= -1.31) and the MP group (0.01 ±0.01 mV; *t*(9)=-9.1, *p*<0.01, *Hedges’s g*= -2.6) suggesting that cerebellar stimulations alone did not induce descending volleys at the cervicomedullary junction.

***Rest motor threshold, MEP_target_, and CS intensity***

We used independent t-tests to ensure that Control and MP groups were not statistically different regarding the rest motor threshold (rMT), the MEP_target_ amplitude, and the cerebellar stimulation (CS) intensity. The rMT (MP group: 42.5 ±6.93 %MSO, Control group: 43.9 ±6.58 %MSO), the MEP_target_ amplitude (MP group: 0.47 ±0.17 mV, Control group: 0.56 ±0.33 mV), and the CS intensity (MP group: 65.3 ±9 %MSO, Control group: 65.9 ± 9.94 %MSO) were not statistically different between groups (all *p’s*>0.1).

***Corticospinal excitability (Single coil TMS)***

We used paired sample t-tests to ensure that single-coil TMS intensities at rest remained stable at Post-Test when compared to those at Pre-Test for both groups (MP group, Pre-Test: 52.28 ±9.35 %MSO; Post-Test: 54.75 ±10.31 %MSO, *t*(9)=1.27, *p*=0.23) and Control group, Pre-Test: 54.79 ± 9.73 %MSO; Post-Test: 54.56 ±10.41 %MSO, *t*(9)=0.15, *p*=0.88). This result confirms that an acute session of MP did not significantly increase corticospinal excitability.
